# Supplementary material for: Modeling glioblastoma heterogeneity as a dynamic network of cell states
Source: Mol Syst Biol. 2021 Sep 16;17(9):e10105. doi: 10.15252/msb.202010105 (PMC8444284; doi:10.15252/msb.202010105)
Supplement: Supplementary file 6 — Source Data for Figure 5 [file MSB-17-e10105-s004.zip › Figure5A_sourcedata/GSEA_3017/nosigdb_stateB.GseaPreranked.1622795021725/DOROSZKO_U3054MG_CLUSTER_4.html]

Details for gene set DOROSZKO\_U3054MG\_CLUSTER\_4[GSEA]

|  || Dataset | state43017 |
| Phenotype | NoPhenotypeAvailable |
| Upregulated in class | na\_pos |
| GeneSet | DOROSZKO\_U3054MG\_CLUSTER\_4 |
| Enrichment Score (ES) | 0.5355753 |
| Normalized Enrichment Score (NES) | 3.414897 |
| Nominal p-value | 0.0 |
| FDR q-value | 0.0 |
| FWER p-Value | 0.0 |
Table: GSEA Results Summary

  

Fig 1: Enrichment plot: DOROSZKO\_U3054MG\_CLUSTER\_4      
 Profile of the Running ES Score & Positions of GeneSet Members on the Rank Ordered List

  

| PROBE | GENE SYMBOL | GENE\_TITLE | RANK IN GENE LIST | RANK METRIC SCORE | RUNNING ES | CORE ENRICHMENT || 1 | HIST1H4C |  |  | 0 | 1.373 | 0.0107 | Yes |
| 2 | HIST1H1B |  |  | 1 | 1.272 | 0.0206 | Yes |
| 3 | HIST1H1D |  |  | 2 | 1.180 | 0.0298 | Yes |
| 4 | TOP2A |  |  | 3 | 1.079 | 0.0382 | Yes |
| 5 | HIST1H1E |  |  | 5 | 0.983 | 0.0437 | Yes |
| 6 | RRM2 |  |  | 7 | 0.875 | 0.0484 | Yes |
| 7 | FAM111A |  |  | 10 | 0.813 | 0.0505 | Yes |
| 8 | UBE2C |  |  | 11 | 0.802 | 0.0568 | Yes |
| 9 | TPX2 |  |  | 12 | 0.801 | 0.0630 | Yes |
| 10 | NUSAP1 |  |  | 13 | 0.801 | 0.0693 | Yes |
| 11 | HMGB2 |  |  | 14 | 0.786 | 0.0754 | Yes |
| 12 | FBXO5 |  |  | 15 | 0.785 | 0.0815 | Yes |
| 13 | NCAPG |  |  | 16 | 0.778 | 0.0875 | Yes |
| 14 | SPC25 |  |  | 17 | 0.763 | 0.0935 | Yes |
| 15 | PRC1 |  |  | 18 | 0.756 | 0.0994 | Yes |
| 16 | ESCO2 |  |  | 20 | 0.742 | 0.1030 | Yes |
| 17 | TACC3 |  |  | 21 | 0.741 | 0.1088 | Yes |
| 18 | GTSE1 |  |  | 22 | 0.732 | 0.1145 | Yes |
| 19 | HJURP |  |  | 23 | 0.731 | 0.1202 | Yes |
| 20 | KIF23 |  |  | 24 | 0.721 | 0.1258 | Yes |
| 21 | ASPM |  |  | 26 | 0.695 | 0.1291 | Yes |
| 22 | NDC80 |  |  | 27 | 0.695 | 0.1345 | Yes |
| 23 | ATAD2 |  |  | 28 | 0.685 | 0.1399 | Yes |
| 24 | KIF15 |  |  | 29 | 0.684 | 0.1452 | Yes |
| 25 | ARHGAP11A |  |  | 30 | 0.683 | 0.1505 | Yes |
| 26 | PCLAF |  |  | 31 | 0.681 | 0.1558 | Yes |
| 27 | SGO1 |  |  | 32 | 0.677 | 0.1611 | Yes |
| 28 | KPNA2 |  |  | 33 | 0.676 | 0.1663 | Yes |
| 29 | CENPF |  |  | 34 | 0.670 | 0.1715 | Yes |
| 30 | KNL1 |  |  | 35 | 0.666 | 0.1767 | Yes |
| 31 | SGO2 |  |  | 36 | 0.664 | 0.1819 | Yes |
| 32 | KIF4A |  |  | 37 | 0.662 | 0.1871 | Yes |
| 33 | ANLN |  |  | 39 | 0.659 | 0.1901 | Yes |
| 34 | RAD51AP1 |  |  | 40 | 0.657 | 0.1952 | Yes |
| 35 | SMC4 |  |  | 41 | 0.655 | 0.2003 | Yes |
| 36 | MELK |  |  | 42 | 0.655 | 0.2054 | Yes |
| 37 | NUF2 |  |  | 44 | 0.651 | 0.2083 | Yes |
| 38 | PRR11 |  |  | 45 | 0.649 | 0.2134 | Yes |
| 39 | KIFC1 |  |  | 47 | 0.645 | 0.2163 | Yes |
| 40 | DHFR |  |  | 49 | 0.638 | 0.2192 | Yes |
| 41 | NCAPG2 |  |  | 50 | 0.636 | 0.2241 | Yes |
| 42 | CDCA5 |  |  | 52 | 0.632 | 0.2269 | Yes |
| 43 | CKAP2 |  |  | 53 | 0.628 | 0.2318 | Yes |
| 44 | CCNA2 |  |  | 54 | 0.626 | 0.2367 | Yes |
| 45 | FANCI |  |  | 56 | 0.615 | 0.2394 | Yes |
| 46 | MYBL2 |  |  | 58 | 0.612 | 0.2420 | Yes |
| 47 | CKAP2L |  |  | 59 | 0.611 | 0.2468 | Yes |
| 48 | CENPU |  |  | 60 | 0.605 | 0.2515 | Yes |
| 49 | CENPK |  |  | 62 | 0.603 | 0.2541 | Yes |
| 50 | TYMS |  |  | 63 | 0.601 | 0.2588 | Yes |
| 51 | BUB1B |  |  | 64 | 0.596 | 0.2634 | Yes |
| 52 | CCDC34 |  |  | 65 | 0.595 | 0.2680 | Yes |
| 53 | KIF2C |  |  | 66 | 0.591 | 0.2726 | Yes |
| 54 | SMC2 |  |  | 68 | 0.586 | 0.2751 | Yes |
| 55 | CDK1 |  |  | 69 | 0.585 | 0.2796 | Yes |
| 56 | H2AFX |  |  | 71 | 0.576 | 0.2820 | Yes |
| 57 | CKS1B |  |  | 72 | 0.575 | 0.2865 | Yes |
| 58 | CIP2A |  |  | 73 | 0.574 | 0.2910 | Yes |
| 59 | RACGAP1 |  |  | 75 | 0.572 | 0.2933 | Yes |
| 60 | FOXM1 |  |  | 76 | 0.570 | 0.2977 | Yes |
| 61 | MAD2L1 |  |  | 77 | 0.569 | 0.3022 | Yes |
| 62 | DBF4 |  |  | 79 | 0.567 | 0.3045 | Yes |
| 63 | CDCA8 |  |  | 82 | 0.562 | 0.3046 | Yes |
| 64 | CKS2 |  |  | 83 | 0.558 | 0.3090 | Yes |
| 65 | ECT2 |  |  | 84 | 0.558 | 0.3133 | Yes |
| 66 | KIF14 |  |  | 85 | 0.557 | 0.3176 | Yes |
| 67 | E2F7 |  |  | 86 | 0.556 | 0.3220 | Yes |
| 68 | UBE2S |  |  | 87 | 0.555 | 0.3263 | Yes |
| 69 | AURKB |  |  | 89 | 0.548 | 0.3285 | Yes |
| 70 | SPC24 |  |  | 91 | 0.544 | 0.3306 | Yes |
| 71 | CENPM |  |  | 92 | 0.544 | 0.3348 | Yes |
| 72 | NCAPH |  |  | 94 | 0.543 | 0.3369 | Yes |
| 73 | PBK |  |  | 95 | 0.540 | 0.3411 | Yes |
| 74 | DEPDC1 |  |  | 97 | 0.536 | 0.3432 | Yes |
| 75 | XRCC2 |  |  | 98 | 0.530 | 0.3473 | Yes |
| 76 | SKA3 |  |  | 99 | 0.525 | 0.3514 | Yes |
| 77 | TMEM106C |  |  | 100 | 0.523 | 0.3555 | Yes |
| 78 | RRM1 |  |  | 103 | 0.520 | 0.3553 | Yes |
| 79 | MXD3 |  |  | 104 | 0.519 | 0.3594 | Yes |
| 80 | CMC2 |  |  | 105 | 0.516 | 0.3634 | Yes |
| 81 | CDKN3 |  |  | 108 | 0.513 | 0.3632 | Yes |
| 82 | UBE2T |  |  | 110 | 0.512 | 0.3650 | Yes |
| 83 | CDCA3 |  |  | 111 | 0.511 | 0.3690 | Yes |
| 84 | DIAPH3 |  |  | 112 | 0.510 | 0.3730 | Yes |
| 85 | BUB1 |  |  | 113 | 0.508 | 0.3769 | Yes |
| 86 | BIRC5 |  |  | 116 | 0.504 | 0.3766 | Yes |
| 87 | CLSPN |  |  | 117 | 0.503 | 0.3806 | Yes |
| 88 | DTYMK |  |  | 118 | 0.503 | 0.3845 | Yes |
| 89 | CEP152 |  |  | 119 | 0.502 | 0.3884 | Yes |
| 90 | ASF1B |  |  | 120 | 0.501 | 0.3923 | Yes |
| 91 | PSMC3IP |  |  | 122 | 0.495 | 0.3940 | Yes |
| 92 | KIF18B |  |  | 123 | 0.494 | 0.3979 | Yes |
| 93 | E2F8 |  |  | 125 | 0.493 | 0.3996 | Yes |
| 94 | SPDL1 |  |  | 126 | 0.492 | 0.4034 | Yes |
| 95 | TMPO |  |  | 127 | 0.492 | 0.4072 | Yes |
| 96 | ITGB3BP |  |  | 128 | 0.490 | 0.4111 | Yes |
| 97 | LMNB2 |  |  | 129 | 0.488 | 0.4149 | Yes |
| 98 | SHCBP1 |  |  | 133 | 0.484 | 0.4123 | Yes |
| 99 | GMNN |  |  | 135 | 0.484 | 0.4140 | Yes |
| 100 | NCAPD2 |  |  | 136 | 0.483 | 0.4177 | Yes |
| 101 | FANCB |  |  | 138 | 0.478 | 0.4193 | Yes |
| 102 | CENPL |  |  | 139 | 0.478 | 0.4231 | Yes |
| 103 | C21orf58 |  |  | 142 | 0.470 | 0.4225 | Yes |
| 104 | CDCA4 |  |  | 148 | 0.461 | 0.4155 | Yes |
| 105 | FANCD2 |  |  | 149 | 0.461 | 0.4191 | Yes |
| 106 | ORC6 |  |  | 150 | 0.461 | 0.4227 | Yes |
| 107 | KIF22 |  |  | 152 | 0.459 | 0.4242 | Yes |
| 108 | NCAPD3 |  |  | 153 | 0.458 | 0.4277 | Yes |
| 109 | CDC45 |  |  | 154 | 0.456 | 0.4313 | Yes |
| 110 | SPAG5 |  |  | 156 | 0.455 | 0.4327 | Yes |
| 111 | MCM8 |  |  | 158 | 0.454 | 0.4342 | Yes |
| 112 | CEP78 |  |  | 161 | 0.451 | 0.4335 | Yes |
| 113 | POLQ |  |  | 162 | 0.450 | 0.4370 | Yes |
| 114 | CENPQ |  |  | 163 | 0.446 | 0.4404 | Yes |
| 115 | CDKN2C |  |  | 164 | 0.445 | 0.4439 | Yes |
| 116 | RPA1 |  |  | 165 | 0.445 | 0.4474 | Yes |
| 117 | TK1 |  |  | 167 | 0.444 | 0.4487 | Yes |
| 118 | BRIP1 |  |  | 168 | 0.443 | 0.4522 | Yes |
| 119 | PIMREG |  |  | 169 | 0.443 | 0.4556 | Yes |
| 120 | ATAD5 |  |  | 170 | 0.442 | 0.4590 | Yes |
| 121 | BRCA1 |  |  | 171 | 0.440 | 0.4625 | Yes |
| 122 | CDK5RAP2 |  |  | 173 | 0.438 | 0.4638 | Yes |
| 123 | BRCA2 |  |  | 174 | 0.436 | 0.4672 | Yes |
| 124 | SMCHD1 |  |  | 175 | 0.436 | 0.4706 | Yes |
| 125 | RBBP8 |  |  | 176 | 0.436 | 0.4739 | Yes |
| 126 | MKI67 |  |  | 179 | 0.436 | 0.4731 | Yes |
| 127 | FEN1 |  |  | 180 | 0.435 | 0.4765 | Yes |
| 128 | USP1 |  |  | 185 | 0.430 | 0.4714 | Yes |
| 129 | MIS18BP1 |  |  | 186 | 0.430 | 0.4748 | Yes |
| 130 | DDIAS |  |  | 187 | 0.428 | 0.4781 | Yes |
| 131 | MGME1 |  |  | 188 | 0.428 | 0.4814 | Yes |
| 132 | BARD1 |  |  | 189 | 0.427 | 0.4848 | Yes |
| 133 | HMMR |  |  | 191 | 0.426 | 0.4860 | Yes |
| 134 | MND1 |  |  | 193 | 0.424 | 0.4872 | Yes |
| 135 | MIS18A |  |  | 196 | 0.421 | 0.4862 | Yes |
| 136 | CCDC15 |  |  | 197 | 0.419 | 0.4895 | Yes |
| 137 | MCM4 |  |  | 199 | 0.418 | 0.4906 | Yes |
| 138 | DSN1 |  |  | 202 | 0.413 | 0.4896 | Yes |
| 139 | SYNE2 |  |  | 203 | 0.413 | 0.4928 | Yes |
| 140 | PEG10 |  |  | 206 | 0.410 | 0.4918 | Yes |
| 141 | TCF19 |  |  | 207 | 0.410 | 0.4950 | Yes |
| 142 | NTM |  |  | 209 | 0.409 | 0.4961 | Yes |
| 143 | ZWINT |  |  | 212 | 0.407 | 0.4950 | Yes |
| 144 | LIG1 |  |  | 214 | 0.407 | 0.4961 | Yes |
| 145 | CCNE2 |  |  | 215 | 0.406 | 0.4992 | Yes |
| 146 | HSPB11 |  |  | 218 | 0.401 | 0.4981 | Yes |
| 147 | MNS1 |  |  | 219 | 0.401 | 0.5012 | Yes |
| 148 | CENPO |  |  | 223 | 0.398 | 0.4980 | Yes |
| 149 | FANCA |  |  | 227 | 0.395 | 0.4948 | Yes |
| 150 | TIMELESS |  |  | 228 | 0.395 | 0.4978 | Yes |
| 151 | MORC4 |  |  | 229 | 0.395 | 0.5009 | Yes |
| 152 | STIL |  |  | 231 | 0.393 | 0.5019 | Yes |
| 153 | PKMYT1 |  |  | 233 | 0.390 | 0.5028 | Yes |
| 154 | POC1A |  |  | 234 | 0.390 | 0.5058 | Yes |
| 155 | EZH2 |  |  | 236 | 0.388 | 0.5067 | Yes |
| 156 | VRK1 |  |  | 238 | 0.388 | 0.5076 | Yes |
| 157 | CDC6 |  |  | 239 | 0.388 | 0.5107 | Yes |
| 158 | PARPBP |  |  | 240 | 0.387 | 0.5137 | Yes |
| 159 | TRIP13 |  |  | 241 | 0.387 | 0.5167 | Yes |
| 160 | PCNA |  |  | 242 | 0.386 | 0.5197 | Yes |
| 161 | POLA1 |  |  | 247 | 0.383 | 0.5142 | Yes |
| 162 | RTKN2 |  |  | 248 | 0.379 | 0.5172 | Yes |
| 163 | CCNB2 |  |  | 250 | 0.379 | 0.5180 | Yes |
| 164 | POLD3 |  |  | 252 | 0.377 | 0.5189 | Yes |
| 165 | MPHOSPH9 |  |  | 254 | 0.375 | 0.5197 | Yes |
| 166 | PLK4 |  |  | 255 | 0.374 | 0.5226 | Yes |
| 167 | RAD18 |  |  | 256 | 0.373 | 0.5255 | Yes |
| 168 | RFC5 |  |  | 260 | 0.370 | 0.5220 | Yes |
| 169 | CENPJ |  |  | 261 | 0.370 | 0.5249 | Yes |
| 170 | GINS4 |  |  | 265 | 0.367 | 0.5214 | Yes |
| 171 | ASRGL1 |  |  | 266 | 0.366 | 0.5243 | Yes |
| 172 | CENPW |  |  | 267 | 0.366 | 0.5271 | Yes |
| 173 | TUBG1 |  |  | 268 | 0.365 | 0.5300 | Yes |
| 174 | TUBA1C |  |  | 270 | 0.362 | 0.5307 | Yes |
| 175 | SMC1A |  |  | 274 | 0.361 | 0.5272 | Yes |
| 176 | PCNT |  |  | 275 | 0.360 | 0.5300 | Yes |
| 177 | CHAF1A |  |  | 276 | 0.359 | 0.5328 | Yes |
| 178 | MMS22L |  |  | 277 | 0.359 | 0.5356 | Yes |
| 179 | GINS1 |  |  | 283 | 0.357 | 0.5278 | No |
| 180 | PSIP1 |  |  | 284 | 0.357 | 0.5306 | No |
| 181 | SLC2A4RG |  |  | 289 | 0.355 | 0.5249 | No |
| 182 | ZWILCH |  |  | 292 | 0.353 | 0.5234 | No |
| 183 | HAUS8 |  |  | 293 | 0.353 | 0.5262 | No |
| 184 | RFC4 |  |  | 295 | 0.351 | 0.5268 | No |
| 185 | CENPP |  |  | 299 | 0.348 | 0.5232 | No |
| 186 | NSD2 |  |  | 303 | 0.346 | 0.5196 | No |
| 187 | DTL |  |  | 305 | 0.345 | 0.5201 | No |
| 188 | CENPN |  |  | 309 | 0.343 | 0.5165 | No |
| 189 | ABHD3 |  |  | 310 | 0.343 | 0.5191 | No |
| 190 | FAM111B |  |  | 314 | 0.342 | 0.5155 | No |
| 191 | PHF19 |  |  | 319 | 0.339 | 0.5097 | No |
| 192 | CSE1L |  |  | 321 | 0.338 | 0.5102 | No |
| 193 | RFC3 |  |  | 322 | 0.338 | 0.5128 | No |
| 194 | OLFM1 |  |  | 325 | 0.337 | 0.5112 | No |
| 195 | RFC2 |  |  | 331 | 0.333 | 0.5033 | No |
| 196 | MRE11 |  |  | 332 | 0.333 | 0.5059 | No |
| 197 | EMP2 |  |  | 333 | 0.333 | 0.5084 | No |
| 198 | HIRIP3 |  |  | 336 | 0.331 | 0.5068 | No |
| 199 | BTG3 |  |  | 337 | 0.331 | 0.5094 | No |
| 200 | NFATC2IP |  |  | 343 | 0.328 | 0.5014 | No |
| 201 | PHGDH |  |  | 344 | 0.328 | 0.5039 | No |
| 202 | PHTF2 |  |  | 345 | 0.327 | 0.5065 | No |
| 203 | HAT1 |  |  | 352 | 0.324 | 0.4964 | No |
| 204 | TMEM237 |  |  | 353 | 0.324 | 0.4989 | No |
| 205 | CCDC14 |  |  | 357 | 0.322 | 0.4951 | No |
| 206 | LMNB1 |  |  | 363 | 0.321 | 0.4870 | No |
| 207 | XPO1 |  |  | 365 | 0.321 | 0.4874 | No |
| 208 | WDR34 |  |  | 366 | 0.321 | 0.4899 | No |
| 209 | RBBP7 |  |  | 367 | 0.321 | 0.4924 | No |
| 210 | TOPBP1 |  |  | 369 | 0.319 | 0.4928 | No |
| 211 | PARP2 |  |  | 372 | 0.319 | 0.4910 | No |
| 212 | NCAPH2 |  |  | 377 | 0.315 | 0.4850 | No |
| 213 | POLA2 |  |  | 380 | 0.314 | 0.4833 | No |
| 214 | DCK |  |  | 383 | 0.313 | 0.4815 | No |
| 215 | RABL6 |  |  | 387 | 0.312 | 0.4776 | No |
| 216 | PRIM2 |  |  | 391 | 0.311 | 0.4737 | No |
| 217 | HAUS2 |  |  | 393 | 0.311 | 0.4740 | No |
| 218 | TEDC1 |  |  | 399 | 0.309 | 0.4658 | No |
| 219 | PPP1R14C |  |  | 407 | 0.305 | 0.4534 | No |
| 220 | BLM |  |  | 408 | 0.305 | 0.4558 | No |
| 221 | LRR1 |  |  | 410 | 0.304 | 0.4561 | No |
| 222 | PTTG1 |  |  | 413 | 0.302 | 0.4542 | No |
| 223 | ZGRF1 |  |  | 415 | 0.302 | 0.4544 | No |
| 224 | PRADC1 |  |  | 421 | 0.299 | 0.4462 | No |
| 225 | CCNE1 |  |  | 424 | 0.299 | 0.4443 | No |
| 226 | NUP107 |  |  | 430 | 0.298 | 0.4361 | No |
| 227 | SASS6 |  |  | 432 | 0.296 | 0.4363 | No |
| 228 | C12orf75 |  |  | 433 | 0.296 | 0.4386 | No |
| 229 | PHIP |  |  | 440 | 0.295 | 0.4282 | No |
| 230 | TFDP1 |  |  | 444 | 0.294 | 0.4242 | No |
| 231 | MZT1 |  |  | 456 | 0.291 | 0.4032 | No |
| 232 | CBX5 |  |  | 457 | 0.291 | 0.4055 | No |
| 233 | SAE1 |  |  | 463 | 0.289 | 0.3972 | No |
| 234 | RFWD3 |  |  | 464 | 0.289 | 0.3995 | No |
| 235 | NUP155 |  |  | 468 | 0.288 | 0.3954 | No |
| 236 | NUP50 |  |  | 470 | 0.287 | 0.3955 | No |
| 237 | YEATS4 |  |  | 476 | 0.286 | 0.3872 | No |
| 238 | CHEK1 |  |  | 477 | 0.286 | 0.3894 | No |
| 239 | RNASEH2A |  |  | 478 | 0.285 | 0.3916 | No |
| 240 | HADH |  |  | 485 | 0.284 | 0.3812 | No |
| 241 | CDT1 |  |  | 486 | 0.284 | 0.3834 | No |
| 242 | HMGXB4 |  |  | 491 | 0.282 | 0.3772 | No |
| 243 | CHTF18 |  |  | 493 | 0.282 | 0.3772 | No |
| 244 | CTDSPL2 |  |  | 495 | 0.282 | 0.3773 | No |
| 245 | CENPH |  |  | 501 | 0.281 | 0.3690 | No |
| 246 | PSMC3 |  |  | 506 | 0.280 | 0.3627 | No |
| 247 | IKBIP |  |  | 508 | 0.279 | 0.3628 | No |
| 248 | WDHD1 |  |  | 513 | 0.278 | 0.3565 | No |
| 249 | TTF2 |  |  | 514 | 0.278 | 0.3587 | No |
| 250 | DNMT1 |  |  | 523 | 0.277 | 0.3439 | No |
| 251 | SLF1 |  |  | 526 | 0.276 | 0.3419 | No |
| 252 | SAC3D1 |  |  | 529 | 0.276 | 0.3398 | No |
| 253 | FIGNL1 |  |  | 542 | 0.273 | 0.3166 | No |
| 254 | ALYREF |  |  | 546 | 0.273 | 0.3124 | No |
| 255 | ERCC6L |  |  | 557 | 0.271 | 0.2934 | No |
| 256 | DONSON |  |  | 559 | 0.271 | 0.2934 | No |
| 257 | NUP85 |  |  | 563 | 0.270 | 0.2892 | No |
| 258 | GGH |  |  | 564 | 0.270 | 0.2913 | No |
| 259 | CDK2 |  |  | 565 | 0.270 | 0.2934 | No |
| 260 | PPP2R3B |  |  | 566 | 0.270 | 0.2955 | No |
| 261 | DDX11 |  |  | 568 | 0.269 | 0.2955 | No |
| 262 | CTNNAL1 |  |  | 570 | 0.269 | 0.2955 | No |
| 263 | NUP205 |  |  | 571 | 0.269 | 0.2976 | No |
| 264 | ICMT |  |  | 580 | 0.268 | 0.2828 | No |
| 265 | MAP3K20 |  |  | 582 | 0.268 | 0.2827 | No |
| 266 | RPA3 |  |  | 584 | 0.267 | 0.2827 | No |
| 267 | SLC43A3 |  |  | 590 | 0.265 | 0.2742 | No |
| 268 | IDH2 |  |  | 598 | 0.263 | 0.2615 | No |
| 269 | CEP57 |  |  | 605 | 0.262 | 0.2509 | No |
| 270 | RAD1 |  |  | 607 | 0.262 | 0.2508 | No |
| 271 | KDELC2 |  |  | 612 | 0.261 | 0.2444 | No |
| 272 | OXCT1 |  |  | 614 | 0.261 | 0.2444 | No |
| 273 | ZNF519 |  |  | 618 | 0.260 | 0.2401 | No |
| 274 | UBA2 |  |  | 626 | 0.259 | 0.2273 | No |
| 275 | CHCHD3 |  |  | 628 | 0.259 | 0.2272 | No |
| 276 | ARL6IP6 |  |  | 629 | 0.258 | 0.2292 | No |
| 277 | PRPF4 |  |  | 640 | 0.257 | 0.2101 | No |
| 278 | EXOSC8 |  |  | 647 | 0.256 | 0.1994 | No |
| 279 | UHRF1 |  |  | 652 | 0.255 | 0.1930 | No |
| 280 | FN3KRP |  |  | 656 | 0.255 | 0.1887 | No |
| 281 | PIGX |  |  | 657 | 0.255 | 0.1906 | No |
| 282 | PXMP2 |  |  | 663 | 0.253 | 0.1821 | No |
| 283 | THOP1 |  |  | 668 | 0.253 | 0.1756 | No |
| 284 | MYBL1 |  |  | 678 | 0.251 | 0.1586 | No |
| 285 | MIF |  |  | 751 | -0.519 | 0.0107 | No |
| 286 | HES6 |  |  | 752 | -0.523 | 0.0148 | No |
Table: GSEA details [plain text format]

  

Fig 2: DOROSZKO\_U3054MG\_CLUSTER\_4: Random ES distribution      
 Gene set null distribution of ES for **DOROSZKO\_U3054MG\_CLUSTER\_4**
